# The Water to Solute Permeability Ratio Governs the Osmotic Volume Dynamics in Beetroot Vacuoles

**DOI:** 10.3389/fpls.2016.01388

**Published:** 2016-09-15

**Authors:** Victoria Vitali, Moira Sutka, Gabriela Amodeo, Osvaldo Chara, Marcelo Ozu

**Affiliations:** ^1^Departamento de Biodiversidad y Biología Experimental, Facultad de Ciencias Exactas y Naturales, Instituto de Biodiversidad y Biología Experimental y Aplicada, Universidad de Buenos Aires and Consejo Nacional de Investigaciones Científicas y TécnicasBuenos Aires, Argentina; ^2^System Biology Group (SysBio), Institute of Physics of Liquids and Biological Systems (IFLYSIB) CONICET, University of La PlataLa Plata, Argentina; ^3^Center for Information Services and High Performance Computing, Technische Universität DresdenDresden, Germany; ^4^Departamento de Fisiología y Biofísica, Facultad de Medicina, Instituto de Fisiología y Biofísica (IFIBIO–Houssay), Universidad de Buenos Aires y Consejo Nacional de Investigaciones Científicas y TécnicasBuenos Aires, Argentina

**Keywords:** vacuole, red beet, *Beta vulgaris*, water flux, solute flux, gradual gradients, mathematical modeling, simulation

## Abstract

Plant cell vacuoles occupy up to 90% of the cell volume and, beyond their physiological function, are constantly subjected to water and solute exchange. The osmotic flow and vacuole volume dynamics relies on the vacuole membrane -the tonoplast- and its capacity to regulate its permeability to both water and solutes. The osmotic permeability coefficient (*P*_*f*_) is the parameter that better characterizes the water transport when submitted to an osmotic gradient. Usually, *P*_*f*_ determinations are made *in vitro* from the initial rate of volume change, when a fast (almost instantaneous) osmolality change occurs. When aquaporins are present, it is accepted that initial volume changes are only due to water movements. However, in living cells osmotic changes are not necessarily abrupt but gradually imposed. Under these conditions, water flux might not be the only relevant driving force shaping the vacuole volume response. In this study, we quantitatively investigated volume dynamics of isolated *Beta vulgaris* root vacuoles under progressively applied osmotic gradients at different pH, a condition that modifies the tonoplast *P*_*f*_. We followed the vacuole volume changes while simultaneously determining the external osmolality time-courses and analyzing these data with mathematical modeling. Our findings indicate that vacuole volume changes, under progressively applied osmotic gradients, would not depend on the membrane elastic properties, nor on the non-osmotic volume of the vacuole, but on water and solute fluxes across the tonoplast. We found that the volume of the vacuole at the steady state is determined by the ratio of water to solute permeabilites (*P*_*f*_/*P*_*s*_), which in turn is ruled by pH. The dependence of the permeability ratio on pH can be interpreted in terms of the degree of aquaporin inhibition and the consequently solute transport modulation. This is relevant in many plant organs such as root, leaves, cotyledons, or stems that perform extensive rhythmic growth movements, which very likely involve considerable cell volume changes within seconds to hours.

## Introduction

Plant cell vacuoles are involved in multiple functions such as the storage of sugars, proteins, aminoacids and pigments (Neuhaus, 2007; Pourcel et al., 2010; Martinoia et al., 2012), isolation of toxic and xenobiotic compounds (Wink, 1997), and defense processes (Mauch and Staehelin, 1989). However, vacuoles are considered critical in the maintenance of cell volume and turgor (Matile, 1978) by tuning up water and solutes fluxes (De, 2000; MacRobbie, 2006) while sustaining the cytosolic pH (Kulichikhin et al., 2009). Thus, it could be expected that vacuole volume is a direct consequence of the water transport across the tonoplast, which in turn would be osmotically affected by the solute transport. On the other hand, it is conceivable that the vacuole could involve regions not participating in the osmotic response, implying that the concomitant osmotic fluxes could be lower than expected. Additionally, tonoplast elastic properties could limit the vacuole volume as well. How water exchange, solutes transport, non-osmotic volume and elastic properties affect volume vacuole changes have not been completely elucidated yet.

**Figure 1:**
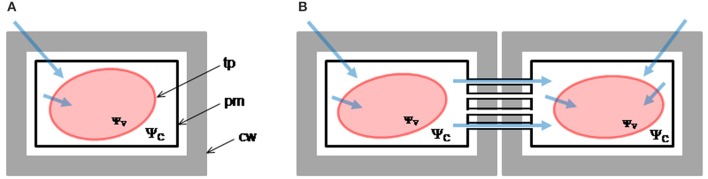
**Water pathways in plant cells**. Light blue arrows schematize the water pathways in isolated **(A)** and connected cells **(B)**. In isolated cells, water entering the vacuole moves through the cell wall (cw) and the biomembranes, i.e., the plasma membrane (pm) and the tonoplast (tp). In addition to these pathways, in connected cells water can pass from one cell to the adjacent neighbor through simplastic pathway. Here, the impact of the imposed osmotic gradient is likely to be more attenuated. The difference of magnitude between the water potential of the cytoplasm (ψ_C_) and the vacuole (ψ_V_) mediates turgor maintenance (ψ_C_ >> ψ_V_).

The osmotic permeability coefficient (*P*_*f*_) is the parameter that better characterizes the capability of the membrane to transport water when it is submitted to an osmotic gradient. In plants, *P*_*f*_ values were reported both in protoplast as in isolated vacuoles of different species (Morillon and Lassalles, 1999; Amodeo et al., 2002; Murai-Hatano and Kuwagata, 2007). Usually, *P*_*f*_ is determined from the initial rate of volume change, when the surrounding medium switches, almost instantaneously, from iso-osmotic to aniso-osmotic. Under physiological conditions, the volume kinetics depends on the cell type. Pulvinar motor cells of *Mimosa pudica* shrink up to 25% within seconds (Fleurat-Lessard et al., 1997), while in guard cells of different species volume can rise up to 40% within minutes (Franks et al., 2001). Moreover, many plant organs such as leaves, cotyledons or stems, show rhythmic growth movements that involve considerable volume changes in nonspecialized cells (Jarillo et al., 2001; Siefritz et al., 2004), in longer time scales -tens of minutes to hours- (Moshelion et al., 2004). The gradual modification of the steady-volume in plant cells, or organelles following slight changes in the osmotic gradient, contrasts with the almost instantaneous osmolality variations imposed during *P*_*f*_ measurement. This could also be the case for cells connected via plasmodesmata (Figure 1), where the passive flow between cytosols is also crucial for turgor changes (Volkov, 2015).

Cells of growing tissues require a considerable supply of water; thus, the hydraulic conductivity is high in these cells (Cosgrove and Steudle, 1981; Steudle and Boyer, 1985). In this way, the vacuoles of such cells present high expression of aquaporins, as γ-TIP (Chrispeels and Maurel, 1994). Gating mechanisms mediated by pH were thoroughly studied in certain plant aquaporins (Tournaire-Roux et al., 2003; Törnroth-Horsefield et al., 2006; Soto et al., 2010; Leitaõ et al., 2012; Frick et al., 2013). In isolated vacuoles from *Beta vulgaris* an inhibitory effect of pH on aquaporins at the tonoplast level—vacuoles and vesicles—was reported (Amodeo et al., 2002; Sutka et al., 2005). In addition, the steady state volume is different under different pH conditions (Amodeo et al., 2002).

In this study, we quantitatively investigated the volume dynamics of *B. vulgaris* vacuoles when exposed to both progressive osmotic gradients and changes in the environmental pH. We tested five hypotheses, either vacuole volume changes are controlled by (1) water transport alone, (2) water and solute transport, (3) water transport together with mechanical tension of the vacuole membrane and (1) and (2) in the presence of a non-osmotic vacuole volume. To test the plausibility of these hypotheses, we encoded them in mathematical models that were contrasted against the obtained experimental data on vacuole volume and external osmolality time courses.

## Model description

### Modeling the osmotic response in beetroot vacuoles

Following the seminal work of Kedem and Katchalsky (1958), mathematical models were proposed in order to study the water transport processes across cell membranes. In the animal kingdom, most of these models were developed to understand mechanisms related with cell volume regulation (Hernandez and Cristina, 1998; Lucio et al., 2003; for a review see Chara et al., 2011) and water channels (Pickard, 2008). In the plant kingdom, a mathematical model of turgor pressure relaxations was developed based on the contribution of water and solutes transport and the cell wall elasticity in algae cells from *Chara coralline* (Wendler and Zimmermann, 1985). A variant of this model (restricted to water transport) was applied to cortical cells of wheat roots (Zhang and Tyerman, 1999). This idea of restricting a mathematical model to the study of water transport across the tonoplast and the plasma membrane was applied later based on the experimental information obtained only from protoplasts (Moshelion et al., 2004; Kuwagata and Murai-Hatano, 2007; Sommer et al., 2007).

**Figure 2:**
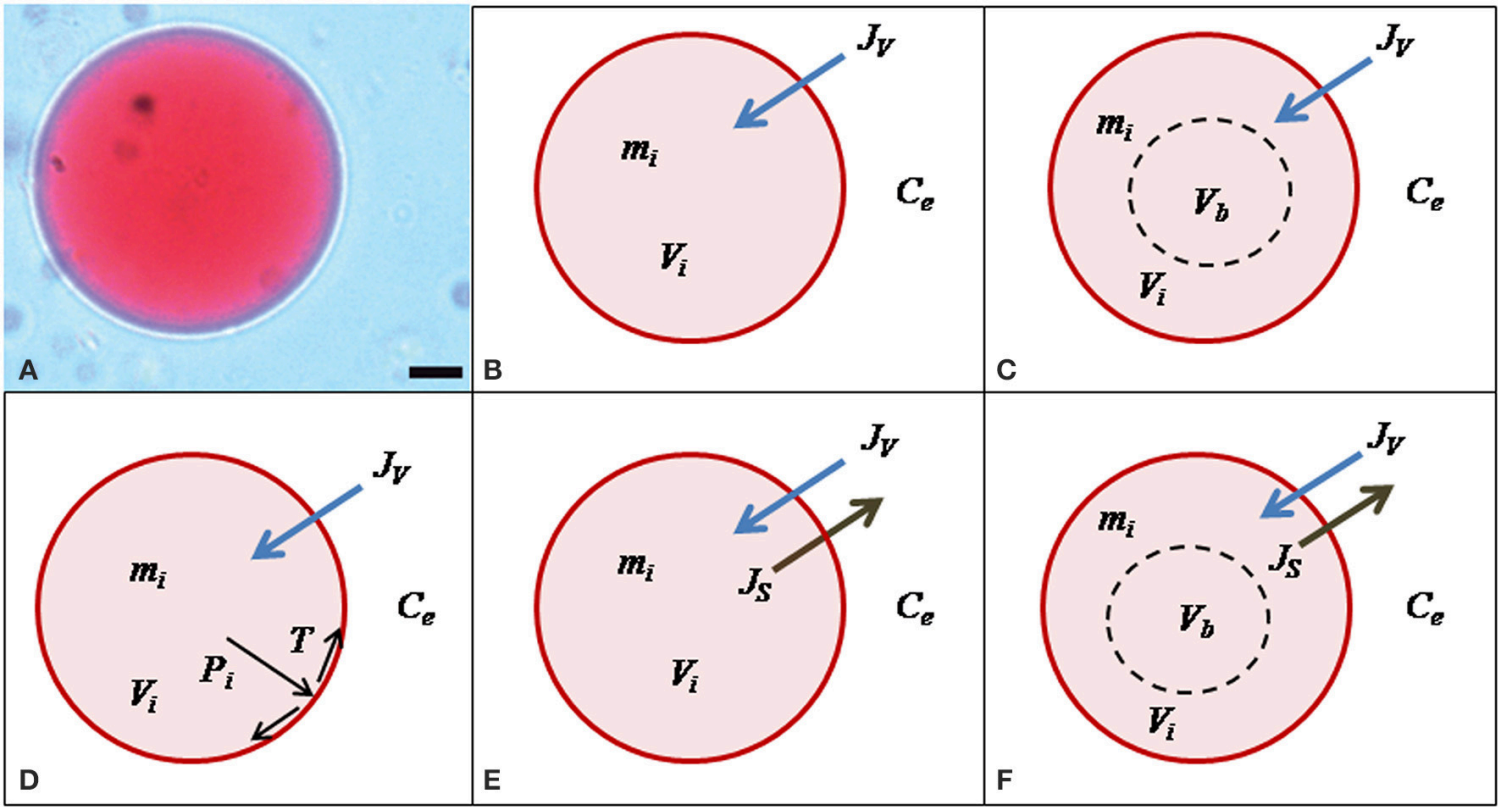
**Mathematical models of vacuole volume dynamics. (A)** Microscopic image of a single *Beta vulgaris* root vacuole under iso-osmotic conditions; scale bar = 10 μm. **(B)** The *W* model assumes that only water moves across the membrane of the vacuole, thus the only parameter is *P*_*f*_. **(C)** The *WNOV* model also considers that only water moves across the membrane but part of the vacuole volume is not involved in the osmotic response (*V*_*b*_). This model also has two parameters: *P*_*f*_ and *V*_*b*_. **(D)** The general model for water flow considers that *J*_*V*_ can be consequence of the combined effects of osmotic (Δ*osm*) and hydrostatic (Δ*P*) driving forces. In the *WME* model, Δ*P* was determined by the elastic modulus of the membrane (ε) and the volume change (Δ*V*) of the vacuole, in a hypo-osmotic medium. This model has two parameters: *P*_*f*_ and ε. **(E)** The *WS* model considers that water and solute fluxes (*J*_*V*_ and *J*_*s*_, respectively) are driven by the osmotic gradient (Δ*osm*) and all the volume of the vacuole (*V*_*i*_) is active. This model has two parameters: *P*_*f*_ and *P*_*s*_**. **(F)** The *WSNOV* model considers that besides the inactive volume *V*_*b*_ the internal mass of solutes (*m*_*i*_) can be modified by the solute flux (*J*_*s*_). This model has three parameters: *P*_*f*_, *P*_*s*_, and *V*_*b*_**. For a detailed description of these models see the text.

We encoded five different hypotheses in mathematical models that were contrasted against the here obtained experimental data on vacuole volume and external osmolality time-courses as well as data from the literature (Amodeo et al., 2002). All these models are schematically shown in Figure 2. For clarity, the *W* model is described first and the specific modifications for the other models are indicated later. Variables and parameters of the models, as well as initial conditions are presented in Table S1.

#### The water model

Our approach started by considering the simplest hypothesis: vacuole volume dynamics depends only on water movement across the vacuole membrane. This hypothesis is encoded in the *Water Model* (*W*) and has only one free parameter: the water osmotic permeability coefficient (*P*_*f*_).

**Figure 3:**
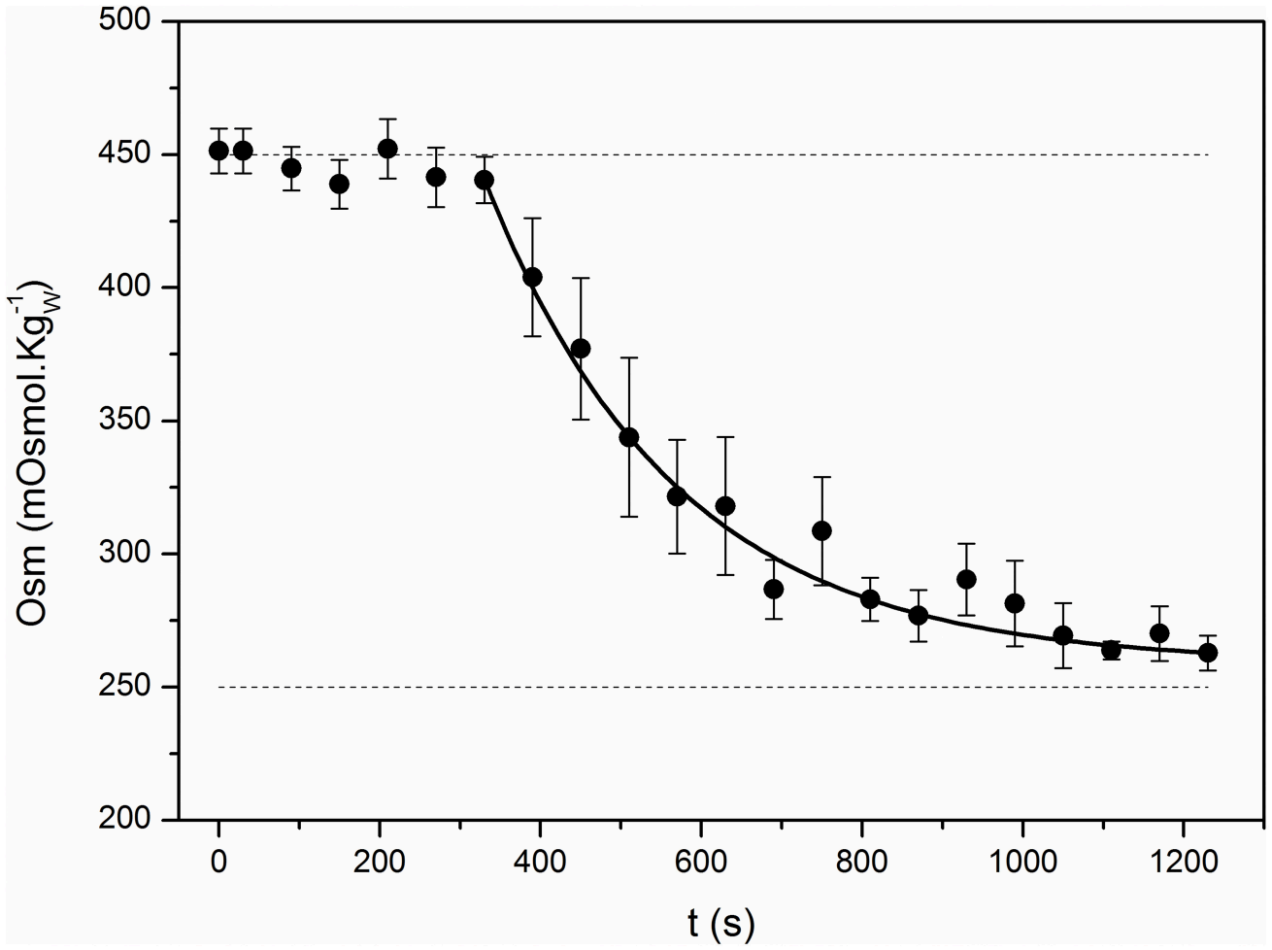
**Time course of the external osmolality**. The kinetics of osmolality in the external chamber was registered in control experiments. The iso-osmotic condition (450 mOsmol.${\text{Kg}}_{\text{w}}^{-1}$) was maintained during 300 s and then, the external solution was replaced for a hypo-osmotic one (260 mOsmol.${\text{Kg}}_{\text{w}}^{-1}$). Small aliquots (50 μL) were regularly taken from the external chamber to measure osmolality along time. Black symbols represent the mean ± SEM of 7 independent experiments. Continuous line shows the fitting to an exponential function $\left(C_{e}\left(t\right)=B\cdot e^{\left(-t/t*\right)}+C_{e}*\right)$. The fitting parameters are: *B* = *731.5* ± *94.5* mOsmol.${\text{Kg}}_{\text{w}}^{-1}$; t^*^ = *237.2* ± *22.4* s; and *C*_e_^*^ = *258.8* ± *3.2* mOsmol.${\text{Kg}}_{\text{w}}^{-1}$; *R*^2^ > 0.98).

The following assumptions are considered:

1. The vacuoles are assumed to be spheres of a certain volume *V* (cm^3^) and surface area *A* (cm^2^). The area *A* was calculated in each iteration from the corresponding *V*-value. However, assuming that *A* is a constant does not change the results (Figure S1).
2. Isolation of vacuoles leads to intra-vacuolar potassium depletion because of the applied gradients (Amodeo et al., 2002). In addition, the membrane potential of isolated vacuoles was probed to be closed to 0 mV (Alexandre et al., 1986). Therefore, in this scenario, and since ions are absent in the solutions used in our experiments, then modulation of solute fluxes by membrane potential changes can be neglected.
3. In the initial condition, the vacuole was equilibrated with an iso-osmotic extracellular compartment with an osmolality *C*_0_, and then exposed to a hypo-osmotic solution with osmolality *C*_*e*_.
4. Since the external solution was perfused into the experimental chamber, the time course of *C*_*e*_ was experimentally determined (Figure 3) and fitted to a single exponential function:
  (1) $$\begin{array}{r} C_{e}\left(t\right)=B\cdot e^{\left(-t/t^{*}\right)}+C_{e}^{*} \end{array}$$
  Where *B* = *731.5* ± *94.5* mOsmol.${\text{Kg}}_{\text{w}}^{-1}$; t^*^ = *237.2* ± *22.4* s; and $C_{e}^{*}$ = *258.8* ± *3.2* mOsmol.${\text{Kg}}_{\text{w}}^{-1}$. *B*: the amplitude of the single exponential function.
5. Water is transported across the tonoplast (which includes the lipid bilayer and aquaporins) according to the phenomenological law of osmosis:
  (2) $$\begin{array}{l} J_{V}\left(t\right)=A\cdot P_{f}\cdot V_{W}\cdot \left(\frac{M_{0}}{V\left(t\right)}-C_{e}\left(t\right)\right) \end{array}$$
  Where *J*_*v*_ is the osmotic volume flux across the tonoplast (cm^3^.s^−1^), *V*, *A*, *P*_*f*_, and *C*_*e*_*(t)* are the vacuole volume (cm^3^), the surface area (cm^2^), the osmotic permeability coefficient (cm.s^−1^) of the tonoplast, and the extracellular osmolality (mOsmol.${\text{Kg}}_{\text{w}}^{-1}$), respectively. For unit consistency, the osmolality was converted to mol.cm^−3^ in simulation routines. *V*_*w*_ represents the partial molar volume of water (18 cm^3^.mol^−1^). It is assumed that the volume flux is composed mainly of water, an assumption that is implicitly based on the idea that the solution transported is highly diluted. The total number of moles of the intracellular solute species is represented by the initial mass of the vacuole (*M*_0_), which is determined by the initial vacuole concentration (*C*_0_) and volume (*V*_0_). This model assumes that mass flux is zero, then *M*_0_ is constant.
6. The rate of volume change is affected only by the osmotic volume flux, i.e., the continuity theorem is fulfilled:
  (3) $$\begin{array}{l} \frac{dV}{dt}=J_{V} \end{array}$$

#### Specific modifications for the other models

##### The water and non-osmotic volume model

A more complex model was hypothesized and tested in protoplasts (Sommer et al., 2007) considering that not all the protoplast volume is osmotically active. Based on this hypothesis, we here developed a model to be tested in isolated vacuoles, the *Water and Non-Osmotic Volume Model* (*WNOV*), which has two free parameters: the water osmotic permeability coefficient (*P*_*f*_) and the non-osmotic volume (*V*_*b*_).

The total volume of the vacuole is *V(t)* = *V*_*i*_*(t)*+*V*_*b*_, where *V*_*i*_*(t)* is the osmotic volume. Therefore, Equation (2) is modified according to:

(4) $$\begin{array}{l} J_{V}\left(t\right)=A\cdot P_{f}\cdot V_{W}\cdot \left(\frac{M_{0}}{V\left(t\right)-V_{b}}-C_{e}\left(t\right)\right) \end{array}$$

As well as in the *W* model, *M*_0_ is constant.

##### The water and membrane elasticity model

Our third hypothesis considers the effect of the internal pressure of the vacuole (as consequence of the water influx) on the tonoplast tension. This hypothesis is encoded in the *Water and Membrane Elasticity Model* (*WME*). In this model, the vacuole volume is determined by the combined effects of osmosis and membrane mechanics and has two free parameters: the water osmotic permeability coefficient (*P*_*f*_) and the volumetric elastic modulus of the tonoplast (ε).

1. The general diffusion model for water (Equation 5) involves two possible driving forces to generate water movements (Finkelstein, 1987). These are the osmotic difference and the hydrostatic pressure difference (Δ*P*).
  (5) $$\begin{array}{l} J_{V}\left(t\right)=A\cdot P_{f}\cdot V_{W}\cdot \left(\Delta C-\frac{\Delta P}{RT}\right) \end{array}$$
  Where, *R* is the universal gas constant (8.31 × 10^7^ dyn.cm.K^−1^.mol^−1^) and *T* is the absolute temperature in the perfusion experiments (293 K); Δ*C* and Δ*P* are detailed below.
2. As in Eq. 2, Δ*C* is defined as $\frac{M_{0}}{V\left(t\right)}-C_{e}.$
3. Δ*P* (dyn.cm^−2^) was calculated from the volumetric elastic modulus ε (dyn.cm^−2^), defined as (Cosgrove and Steudle, 1981; Ozu et al., 2013):
  (6) $$\begin{array}{l} \varepsilon =V\cdot \frac{\Delta P}{\Delta V} \end{array}$$
  Where the pressure-volume ratio is an elastic coefficient ε^*^:
  (7) $$\begin{array}{l} \varepsilon^{*}=\frac{\Delta P}{\Delta V} \end{array}$$
  Then, the hydrostatic pressure change (Δ*P*) induced by water entering into the vacuole, and the volumetric elastic modulus (ε) were calculated in each iteration step according to:
  (8) $$\begin{array}{r} \Delta P=\varepsilon^{*}\cdot \left(V\left(t\right)-V_{0}\right) \end{array}$$
  (9) $$\begin{array}{r} \varepsilon =V\left(t\right)\cdot \varepsilon^{*} \end{array}$$
  Equations (5) and (8) replace Equation 2 and were used to simulate the osmotic response and find the *P*_*f*_ and ε (dyn.cm^−2^) values (with Equation 9) that showed the best fit to experimental data. For better comparison to reported values, ε is present in MPa (Table S4).

##### The water and solute model

An alternative hypothesis considers that the vacuole volume would be determined by the combined effects of water and solute fluxes; for example, water entering the vacuole and solutes moving simultaneously across the tonoplast in the opposite way, i.e., leaving the vacuole. This is the *Water and Solute Model* (*WS*) which has two free parameters: the water osmotic permeability coefficient (*P*_*f*_) and the solute permeability coefficient (*P*_*s*_). *P*_*s*_ refers to a generic permeability of all the possible solutes involved in the transport processes across the tonoplast.

1. The solute can be transported across the membrane by simple diffusion (Kedem and Katchalsky, 1958):
  (10) $$\begin{array}{l} J_{S}\left(t\right)=-A\cdot P_{S}\cdot \left(\frac{M\left(t\right)}{V\left(t\right)}-C_{e}\left(t\right)\right) \end{array}$$
  Where *J*_*S*_ is the solute flux across the membrane (cm^3^.s^−1^), while *P*_*S*_ is the solute permeability of this membrane (cm.s^−1^). The minus symbol indicates the opposite direction for solute flux compared to water flow.
2. The dynamics of intra-vacuole solutes is determined by the transport across the tonoplast:
  (11) $$\begin{array}{l} \frac{dM}{dt}=J_{S} \end{array}$$
3. The interactions between the electrical potential of the membrane and both the vacuole volume and the solute flux across the tonoplast are neglected, according to the arguments exposed in the point (2) of the *W* model.
4. From the previous assumptions, the following differential equations can be obtained:
  (12) $$\begin{array}{l} \frac{dV}{dt}=A\cdot P_{f}\cdot V_{W}\cdot \left(\frac{M\left(t\right)}{V\left(t\right)}-C_{e}\left(t\right)\right) \end{array}$$
  (13) $$\begin{array}{l} \frac{dM}{dt}=-A\cdot P_{S}\cdot \left(\frac{M\left(t\right)}{V\left(t\right)}-C_{e}\left(t\right)\right) \end{array}$$
  Hence, Equations (12) and (13), along with the initial conditions *V(t* = *0)* = *V*_0_ and *M(t* = *0)* = *M*_0_, essentially describe the dynamics of water and solute transport by osmosis and diffusion across the tonoplast. While *V*_0_ is experimentally determined by video-microscopy, *M*_0_ is calculated from *V*_0_ and the measured osmolality *C*_0_ at which the vacuoles were initially exposed in all the experiments (*M*_0_ = *V*_0_.*C*_0_). Then, *P*_*f*_ and *P*_*S*_ are the two free parameters of the model. Although simultaneous water and solute transport is considered, this model does not consider interactions between water and solutes; neither whether the pathways are shared or not (Kedem and Katchalsky, 1958).

##### The water solute and non-osmotic volume model

The vacuole volume could also be affected by the combined effects of water and solute fluxes in a fraction of the vacuole volume. This model is named the *Water, Solute and Non-Osmotic Volume Model* (*WSNOV*) and has three free parameters: the water osmotic permeability coefficient (*P*_*f*_), the solute permeability coefficient (*P*_*s*_) and the non-osmotic volume (*V*_*b*_).

As in the *WNOV* model, *V(t)* = *V*_*i*_*(t)* + *V*_*b*_. Therefore, Equations (6) and (7) are replaced by:

(14) $$\begin{array}{r} \frac{dV}{dt}=A\cdot P_{f}\cdot V_{W}\cdot \left(\frac{M\left(t\right)}{V\left(t\right)-V_{b}}-C_{e}\left(t\right)\right) \end{array}$$

(15) $$\begin{array}{r} \frac{dM}{dt}=-A\cdot P_{S}\cdot \left(\frac{M\left(t\right)}{V\left(t\right)-V_{b}}-C_{e}\left(t\right)\right) \end{array}$$

## Materials and methods

### Plant material and isolation of vacuoles from red beetroots

*B. vulgaris* plants were grown in the field, harvested after 90 days, transferred to the laboratory and maintained under semi-controlled conditions until use. Vacuoles were mechanically isolated as previously described (Amodeo et al., 2002). Briefly, the storage roots were cut in 1 cm^3^ sections and incubated during 20 min in a 2 M sucrose solution. These sections were collected after removing the excess solution with tissue paper, and transferred to a control solution containing 400 mM mannitol, 1 mM EDTA, 20 mM Tris-Mes, at pH 7.6, and a final osmolality of 450 mOsm.${\text{Kg}}_{\text{w}}^{-1}$. The slices were finely chopped using a sharp razor blade and the vacuoles were released in the medium.

### Vacuole volume changes studied by video-microscopy in perfusion experiments

Perfusion experiments were performed as described (Amodeo et al., 2002). In these experiments, an aliquot of the solution containing vacuoles was transferred to a small chamber designed *ad*-*hoc*. We developed a perfusion system that allowed us to swap solutions while an individualized vacuole was monitored through time. Initially, the vacuoles were perfused with an iso-osmotic solution (400 mM mannitol, 1 mM EDTA, 20 mM Tris-Mes, pH 7.6, 450 mOsm.${\text{Kg}}_{\text{w}}^{-1}$), which was then switched to a hypo-osmotic one prepared by reducing the final mannitol concentration to 200 mM (final osmolality 260 mOsm.${\text{Kg}}_{\text{w}}^{-1}$).

In order to test the effect of different proton concentrations on the volume of the vacuoles, they were perfused with the above mentioned solutions with modifying buffer pH to final concentrations of 7.0 and 6.8, as was previously published for pH 8.6, 7.6, and 6.6 (Amodeo et al., 2002).

In order to validate the results obtained with the *WS* model, we also performed perfusion experiments under hyper-osmotic conditions and pH 7.6 (Figure S4). In these experiments, the iso-osmotic solution was switched to a hyper-osmotic one by increasing the mannitol concentration to 660 mOsm.${\text{Kg}}_{\text{w}}^{-1}$.

#### Video-microscopy set-up

Time course of the relative volume change of isolated vacuoles suspended in the medium was followed by video-microscopy. Vacuoles were observed by transmitted light using 300X magnification in an inverted Olympus IMT-2 microscope connected with a digital video camera (Electrim EDC-1000, EBSCO, USA). The images were digitalized through a PC acquisition board (total amplification 1300X). Single vacuole images were recorded every 30 s during 10 min after changing the solution. Vacuole diameters were measured from the stored images employing commercial image software (Optimetric 1.0; Bioscan, USA), and a calibrated microscope slide was employed to convert image diameters in pixels to real metric units. The scaling factor was 2.98–3.50 μm.pixels^−1^ so it was not significant compared with the uncertainty coming from the obtained values in simulated routines.

### Determination of the external osmolality time course when changing the external solution

Control experiments were done in order to determine the time course of the external osmolality (Figure 3). The experimental chamber was loaded with the iso-osmotic solution (450 mOsm.${\text{Kg}}_{\text{w}}^{-1}$) and after 300 s the solution was replaced by the hypo-osmotic solution (260 mOsm.${\text{Kg}}_{\text{w}}^{-1}$). From the beginning of the experiment, aliquot samples were taken every 30 s to determine osmolality with a vapor pressure osmometer (VAPRO 5520, Wesco, Logan, UT). Results from 7 independent experiments were fitted to exponential functions. The mean of these functions was introduced in the model detailed below to describe the external osmolality time course.

The same protocol was implemented when determining the time course of external osmolality in the perfusion experiments with a hyper-osmotic solution (660 mOsm.${\text{Kg}}_{\text{w}}^{-1}$). The results were fitted to an exponential function (Figure S4A), which was then introduced in all the tested models to perform the validation experiments.

### Model-dependent fit to experimental data and further validation

Vacuole volume kinetics was simulated by implementing the models detailed in the Model description section and applying a routine encoded in Visual BASIC. This routine solves the differential equations of the model numerically, and is linked to a fitting routine encoded in the same programming language (software and source code are available by request). In all cases, the program was initially loaded with experimental data of the relative volume time course of the vacuole under hypo-osmotic conditions, estimations of the initial values of the volume and surface of the vacuole, and the internal and external osmolalities. In order to perform the simulations, the differential equations that govern each model were integrated numerically employing the Euler method. An integration step of 0.1 s that guarantees the stability of the numerical method was used.

Algebraically, the problem of fitting consists in the exploration of the parameter space in order to minimize a given fitness function. Here, the fitness function is the sum of the squares of the residuals between the experimental volume of the isolated vacuole and the volume simulated by the model under study at each experimental time step. We called the latter procedure a model-dependent fit in order to emphasize that the constraints imposed by the mathematical model were taken into account during the fitting.

In order to validate results obtained with the tested models we performed simulations assuming hyper-osmotic conditions. For the *WS* model, we used *P*_*f*_ and *P*_*s*_ values from the best fitting ranges obtained from the hypo-osmotic conditions at pH 7.6 (Figure S4B).

### Statistical analysis

Results are presented as mean ± SEM. The Kruskal-Wallis test for multiple comparisons was used followed by the *post-hoc* Mann-Whitney test with the Bonferroni correction (*p* < 0.05).

## Results

### The osmotic response of beetroot vacuoles is reproduced by a model that only depends on the water and solute permeability coefficients

**Figure 4:**
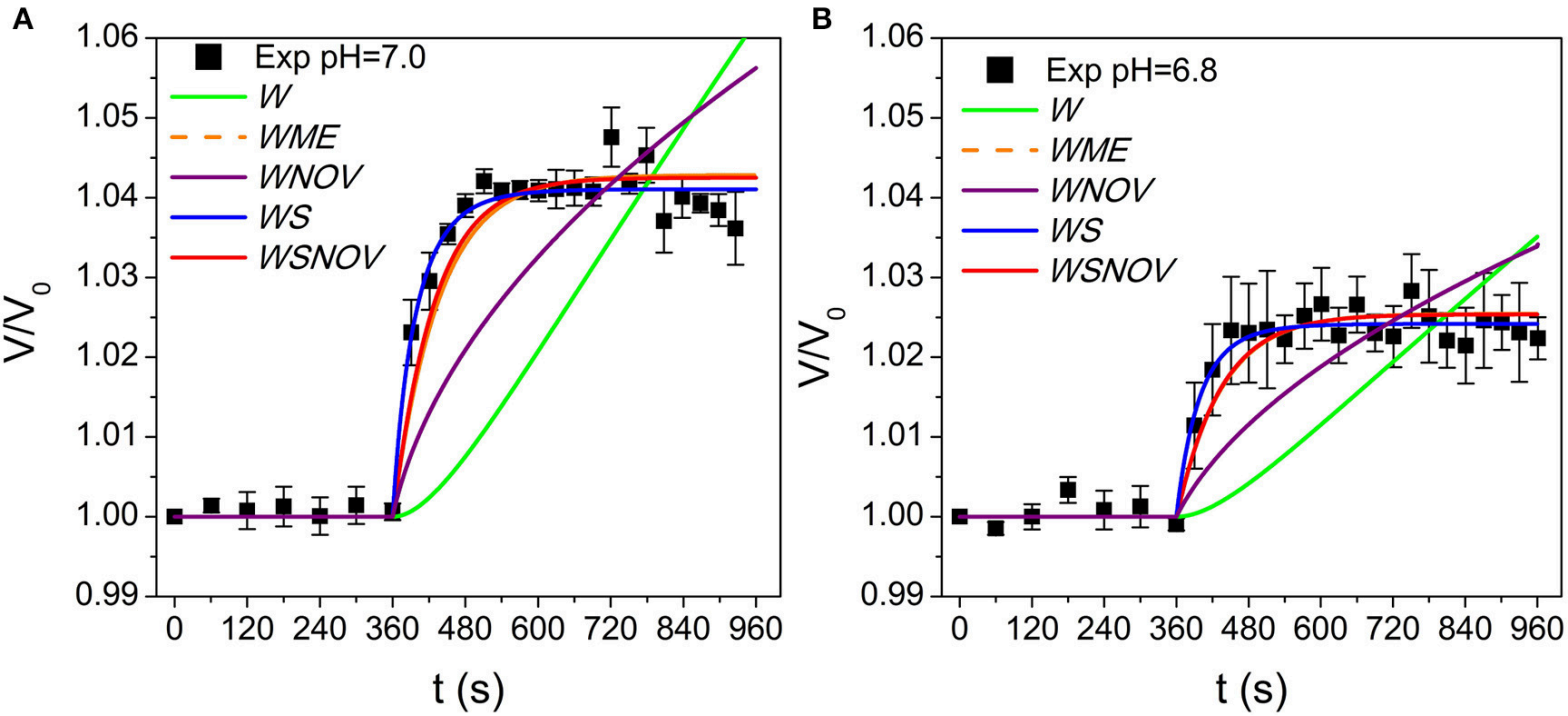
**Model simulations of vacuole volume dynamics fitted to experimental data at pH 7.0 and 6.8. (A)** shows pH 7.0 and **(B)** shows pH 6.8. Simulations obtained with the *W* model and the *WNOV* model cannot reproduce the experimental record. The other three models (*WME, WS*, and *WSNOV*) fit close to experimental data, and are practically not differentiable by visual inspection. Simulations obtained with published data with other pH conditions are shown in Figure S2. The parameter values are shown in Table S4 and the fitting analysis is shown in Tables S2, S3.

To focus on the transport phenomena at the tonoplast level we isolated *B. vulgaris* vacuoles and submitted them to a progressively applied osmotic gradient while monitoring both the external osmolality reduction (Figure 3, black circles) and the increase of the vacuole volume at pH 7.0 (Figure 4A, black squares). We investigated whether the vacuole swelling could be explained by water transport alone, by fitting a mathematical model assuming only water fluxes across the tonoplast (the *W* model, Figure 2B) and simultaneously incorporating a gradual change of osmolality by means of an experimentally determined function (Figure 3, continuous curve). Since the *W* model did not successfully fit to the experimental data (Figure 4A), we concluded that other mechanisms must be considered. A similar result was obtained when fitting a model that also assumes a non-osmotic volume inside the vacuole (the *WNOV* model, Figures 2C, 4A).

In contrast, models assuming simultaneous water and solute fluxes across the tonoplast, with or without non-osmotic volume (the *WSNOV* model in Figure 2F, or the *WS* model in Figure 2E, respectively), successfully fitted to the experimental data (Figure 4A). In addition, a model assuming the tonoplast elasticity counter balancing the volume changes triggered by water fluxes (the *WME* model, Figure 2D) successfully fitted to the experimental record (Figure 4A). All the same results were achieved when reducing pH from 7.0 to 6.8 (Figure 4B).

To discriminate among the three successful models an Akaike's analysis was performed (Table S3). Fitting results indicate that although the *WSNOV* model has three parameters (*P*_*f*_, *P*_*s*_, and *V*_*b*_) it is not a better choice than the other two models. Therefore, this model is discarded by the Ockham's razor principle.

The remaining two models have two parameters (*P*_*f*_ and ε, and *P*_*f*_ and *P*_*s*_), and both of them show similar fitting results (Table S2). However, the analysis shows that the *WS* model is a better choice than the *WME* in all pH conditions tested (Tables S2, S3). Moreover, the best fits obtained with the *WME* model show *P*_*f*_ values that are both too high (Table S4) and do not show the previously reported pH-dependency (Figure 5B from Sutka et al., 2005). Although ε values obtained are slightly higher than those reported in plant cells (Nobel, 2009; Beauzamy et al., 2014), the high *P*_*f*_ values make this model unlikely.

**Figure 5:**
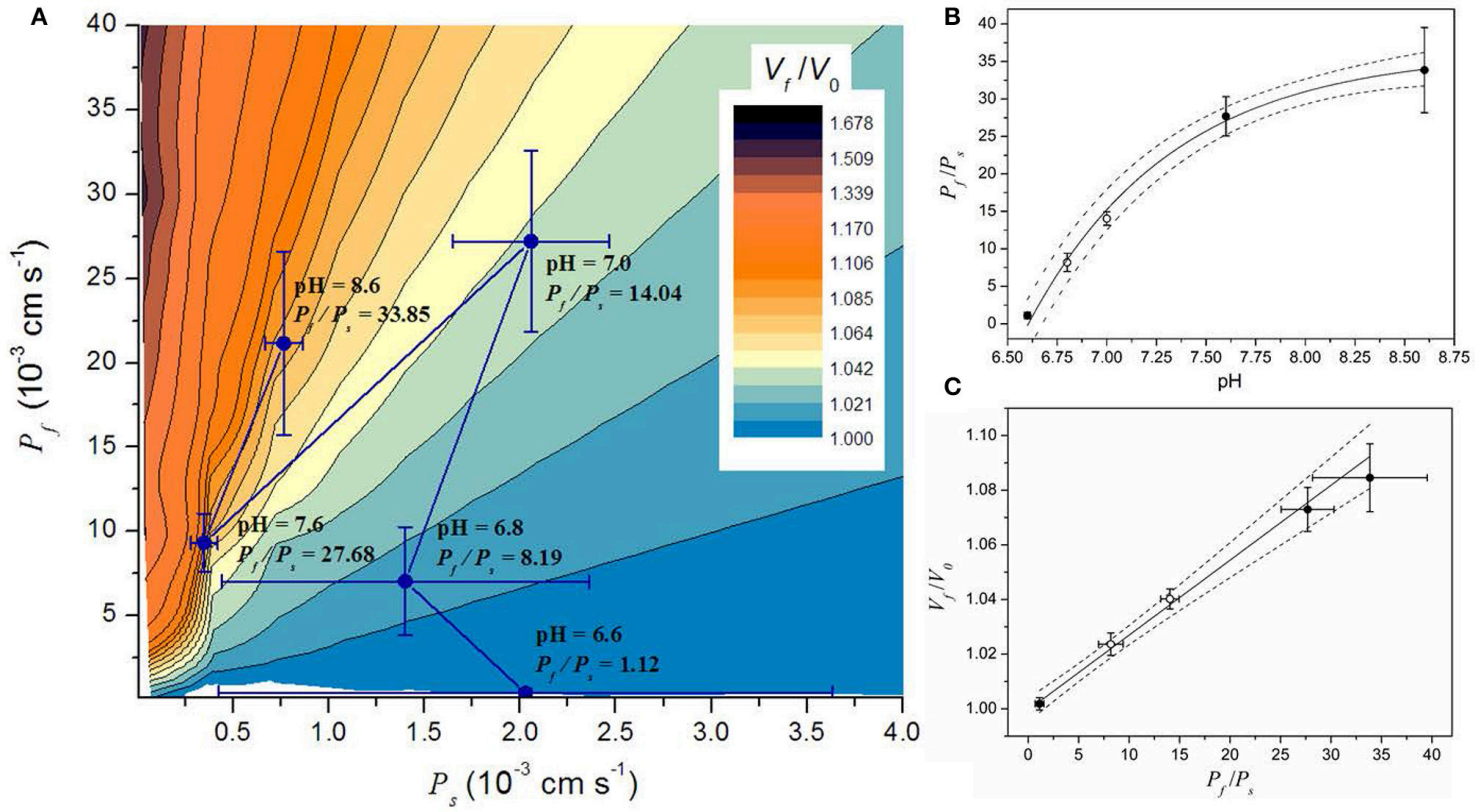
**Simulation results in the context of the parameter space of a model involving water and solutes transport (*WS* model)**. A small portion of the parameter space is shown in **(A)**. The parameter space shows all the possible steady volumes (in color code) predicted by the *WS* model. The *P*_*f*_ and *P*_*s*_ values obtained by fitting simulations are superimposed and shown as mean ± SEM. **(B)** Relationship between the *P*_*f*_*/P*_*s*_ ratio and pH. Symbols represent the mean ± SEM of the *P*_*f*_*/P*_*s*_ ratio. Continuous line shows the fitting to an exponential function $\left(P_{f}/P_{s}\;=A\;\cdot \;e^{\left(-\left(pH-pH*\right)/pH^{\prime }\;\right)}+P_{f}/P_{s}^{*}\right)$. Fitting parameters are: *A* = −36.5 ± 1.4; $P_{f}/P_{s}^{*}$ = 36.3 ± 1.1; pH^*^ = 6.6; and *pH'* = 0.73 ± 0.09; *R*^2^ > 0.99). **(C)** Relationship between the steady volume (*V*_*f*_/*V*_0_) and the *P*_*f*_/*P*_*s*_ ratio. Symbols represent the mean ± SEM of both the *P*_*f*_*/P*_*s*_ and the *V*_*f*_/*V*_0_ ratios obtained with the *WS* model. Continuous line shows the result of a linear fitting (intercept: 1.00 ± 0.002, slope: 0.0025 ± 0.0001, *R*^2^ > 0.99). Dash lines in **(B,C)** represent the 95% CI. In both panels, open circles are data from experiments performed in this work (pH 7.0 and 6.8), and black circles are data taken from previous experiments (Amodeo et al., 2002).

The Akaike's analysis (Tables S2, S3) indicates that the *WS* model is better than the other four models to reproduce the osmotic response of the beetroot vacuoles. Moreover, the best fitting result gave *P*_*f*_ values similar to those experimentally determined by other methodology (Sutka et al., 2005). Table 1 shows the best fitting values obtained for *P*_*f*_ and *P*_*s*_. Although no *P*_*s*_ values were found in literature for comparison, simulations allowed us to obtain the calculated mass flow generated in each iteration by the best fitting *P*_*s*_ value. In the case of vacuoles exposed to pH 7.0 the average mass flow ranges between 6 × 10^6^ and 6 × 10^9^ particles.s^−1^. This result is comparable with transport rates reported in plant cells (Volkov, 2015).

**Table 1:** **Water and Solute permeability coefficients obtained with the *WS* model**.

**pH**	***P_f_* (*10*^−3^*cm.s*^−1^)**	***P_s_* (*10*^−3^*cm.s*^−1^)**	***P_f_*/*P_s_***	***V_f_*/*V*_0_**	***N***
6.6	0.4 ± 0.3	2.0 ± 1.6	1.12 ± 0.44	1.002 ± 0.002	6
6.8	7 ± 3^a^	1.4 ± 0.9	8.19 ± 1.22^e^	1.023 ± 0.004	10
7.0	27 ± 5^b^	2.1 ± 0.4	14.04 ± 0.91^f^	1.040 ± 0.004	14
7.6	9 ± 2^a^	0.35 ± 0.07^c^	27.68 ± 2.62^g^	1.073 ± 0.008	14
8.6	21 ± 5^b^	0.77 ± 0.10^d^	33.85 ± 5.69^g^	1.085 ± 0.012	6

*The water and solute permeability coefficients (P_f_ and P_s_, respectively) are shown as the mean ± SEM of N independent experiments tested with different pH conditions. The P_f_/P_s_ ratio and the steady volume reached in each condition are also shown. Different letters indicate statistically significant differences (p < 0.05, Mann–Whitney-test)*.

In order to test all the models in other pH conditions, fitting simulations were performed with experimental records at pH 8.6, 7.6, and 6.6, which move away from the physiological condition (Figure S2). These experimental data have been previously published (Amodeo et al., 2002) and performed in the same conditions that the new data presented in this work at pH 7.0 and 6.8. In the fitting simulations performed at pH 8.6, 7.6, and 6.6 the *WS* model resulted to be the best one among the five models tested. Altogether, results show that the initial rates of volume change and the steady volumes are different for each pH condition (Table 1 and Figure S3). In addition, results show that the different steady state volumes reached by the vacuoles under different pH conditions cannot be explained only by the *P*_*f*_ decrease.

In order to validate the previous results we performed simulations under hyper-osmotic conditions and contrasted them with experimental records. As well as under hypo-osmotic conditions, the *WS* model resulted to be the best one to reproduce the osmotic experiments (Figure S4A). The *P*_*f*_ and *P*_*s*_ values expected for hyper-osmotic conditions were predicted from the *P*_*f*_ and *P*_*s*_ values ranges, obtained by the fitting simulations performed under hypo-osmotic conditions. All tested models included the function that describes the changes of external osmolality under hyper-osmotic conditions, which was experimentally determined (Figure S4B).

### The steady state volume osmotically reached by beetroot vacuoles depends on the balance between the water and solute permeabilities

By fitting the *WS* model to experimental data at pH 6.8 and 7.0 (both new results presented here) and previously reported data at pH 6.6, 7.6, and 8.6 (Amodeo et al., 2002), we found that the vacuole volume at the steady state is reduced when pH decreases (Figure 4, and Figure S3). To further analysis of this observation, we performed ~1.10^6^ simulations varying *P*_*f*_ and *P*_*s*_ to fully explore the parameter space and evaluate the volume at the steady state (*V*_*f*_/*V*_0_). Figure 5A shows a portion of the parameter space of the *WS* model in terms of *V*_*f*_/*V*_0_ with the best fitting parameter values superimposed. Figure 5A shows that at high *P*_*f*_ values, *P*_*s*_ is restricted to a small range, while at low *P*_*f*_ values *P*_*s*_ can take almost any value. Since the volume increase is governed by the osmotic water flow, results indicate that at low *P*_*f*_ values, for instance when aquaporins are inhibited (Amodeo et al., 2002; Sutka et al., 2005), the solute movement has no effect on volume changes. However, at high *P*_*f*_ values the volume change is strongly dependent on the magnitude of the solute flux conditioned by *P*_*s*_. Inspection of the best fitting parameter values shows that the relationship between the *P*_*f*_/*P*_*s*_ ratio and the pH condition can be fitted to an increasing exponential function (Figure 5B). This monotonic increasing behavior is translated into the steady state volume as result of the water influx into the vacuole that wins the wrestling against the solute efflux, and its consequent water dragging effect. At pH 6.6, the *P*_*f*_/*P*_*s*_ ratio is approximately 1, which can explain why no volume change is observed in this condition. Then, with increasing pH conditions the *P*_*f*_/*P*_*s*_ ratio reaches a maximum asymptotically. In addition, the plot of *V*_*f*_/*V*_0_ vs. the *P*_*f*_/*P*_*s*_ ratio reveals a linear relationship between them (Figure 5C). Altogether, these results show that the volume of the vacuole at the steady state depends on pH due to changes on the *P*_*f*_/*P*_*s*_ ratio.

## Discussion

### Modeling the osmotic response in beetroot vacuoles

A number of mathematical models were previously proposed to simulate water and solute transport across the plasma membrane and tonoplast of plant cells (Wendler and Zimmermann, 1985; Zhang and Tyerman, 1999; Kuwagata and Murai-Hatano, 2007). In this study, we presented a modeling approach, which (1) is focused on the transport processes across the tonoplast and (2) incorporates a progressively applied osmotic gradient, which was experimentally determined. Encoded in this modeling framework, we formulated plausible hypotheses to explain the vacuole volume dynamics. The corresponding mathematical models were contrasted against here presented and previously reported experimental data on the dynamics of *B. vulgaris* root vacuoles.

Since water driven by the osmotic gradient enters the vacuole and produces an increase of turgor pressure, we tested the magnitude of the membrane tension needed to hold the turgor increase. Hence, a model containing a parameter to account for the elastic modulus of the tonoplast (the *WME* model) was here tested. As it was shown in the Results Section, this model was discarded by comparison to the *WS* model. The ε values obtained with the *WME* model are slightly higher than those reported in plant cells (Nobel, 2009; Beauzamy et al., 2014) but are still within the range of the maximal values supported by cells (Peyronnet et al., 2014). However, the *P*_*f*_ values obtained by fitting are too high and do not show the previously reported pH-dependency experimentally observed (Figure 5B from Sutka et al., 2005). The high *P*_*f*_ values would indicate that the water flux needed to generate the pressure difference that balances the osmotic pressure is higher than the possible flux through the tonoplast driven by the given osmotic gradient. This is in accordance with the relationship between osmotic and hydrostatic pressures (Finkelstein, 1987; Ozu et al., 2013).

An alternative hypothesis was postulated on the basis that not all the vacuole volume could be involved in osmosis. It is commonly accepted that there are osmotically inactive components inside the cells, which constitute a non-osmotic volume (*V*_*b*_). This *V*_*b*_ was previously considered in a model tested in protoplasts (Sommer et al., 2007). Although the present study was performed in isolated vacuoles, we tested the contribution of the possible non-osmotic volume. As shown in Table S4, *V*_*b*_ resulted to be 99% of the vacuole volume in the *WNOV* model. These values are higher than reported in literature (Sommer et al., 2007) and this model was discarded by comparison analysis (Tables S2, S3). By other side, a model assuming water and solute transport with non-osmotic volume (the *WSNOV* model) satisfactorily fits to experimental data, with *V*_*b*_ values comparable to those previously reported and discussed by Sommer et al. (2007). However, a model assuming water and solute transport without non-osmotic volume (the *WS* model) resulted to be a better choice in all the conditions tested in this work.

Our results demonstrate that a model governed by the *P*_*f*_ and *P*_*s*_ coefficients fits satisfactorily to the experimental data obtained in isolated vacuoles under different pH conditions, and predicts the hyper-osmotic response in our system. In addition, *P*_*f*_ values obtained with this model agree with those previously reported for vacuoles (Amodeo et al., 2002) or vesicles (Sutka et al., 2005). In the present work, the *P*_*f*_/*P*_*s*_ ratio and the volume of isolated beetroot vacuoles at the steady state were predicted for different pH conditions within a physiological range. In consequence, water and solute fluxes can be calculated by measuring only one of the two permeability coefficients in osmotic experiments under a known pH condition. The relevance of this conclusion gains importance since *P*_*f*_ is easier to be determined than *P*_*s*_, which also gives support to further studies integrating transport of solutes (by means of *P*_*s*_) and water (by means of *P*_*f*_) in vacuoles.

### Relevance of solute movement

As mentioned, the model involving water and solute fluxes (the *WS* model) is the one that better describes the vacuole volume dynamics. Unfortunately, the *P*_*s*_ values obtained with the model could not be experimentally contrasted since, to our knowledge, there are not available data in literature. However, some comparisons and further analysis can be made. According to a recent published analysis, a small plant cell with a surface to volume ratio of 0.9 μm^−1^ might show a transport rate of 2.8 × 10^6^ ions.s^−1^, while a larger plant cell with a surface to volume ratio of 0.16 μm^−1^ has a transport rate of 1.1 × 10^9^ ions.s^−1^ (Volkov, 2015). Although the previous considerations were obtained from ion transport calculations, the solute could be an organic compound without electrical charge. The mass flow obtained in our simulations varied along each experiment from 6 × 10^6^ to 6 × 10^9^ particles.s^−1^, this range is comparable with calculations made in plant cells (Volkov, 2015).

### Relevance of gradual gradients in beetroot

Sucrose plays important roles in plant cell physiology. It is known that vacuoles contain fructose and other organic compounds (Neuhaus, 2007; Ludewig and Flügge, 2013). Evidences from literature report a negative correlation between sucrose and other solutes concentrations (Alexander, 1971). Among these solutes, potassium, sodium, amines, and betaines balance the sucrose concentration (Milford, 2006), e.g., in sugar beet roots sucrose and potassium concentrations are inversely related (Kholodova et al., 1989).

The greatest concentrations of sucrose occur in the cells of the vascular zone. According to the “sucrose gradient hypothesis,” sucrose enters the storage root via the phloem (Giaquinta, 1979), it is transported within the apoplast (Richer and Ehwald, 1983), and then it is stored in the vacuoles of parenchymal cells without hydrolysis (Giaquinta, 1977), where it contributes importantly to turgor pressure (Leigh et al., 1979). The cells closer to the vasculature would have a higher sucrose content because the uptake is linear with the external sucrose concentration over the range 0.5–500 mM (Wyse, 1979). Given this scenario, it is expected that bigger cells have lower sucrose uptake and lower sucrose concentrations because of its location at the end of the diffusive way. In *B. vulgaris*, this hypothesis implies that a differential sucrose concentration is developed in the tissues alongside the diffusive way, being this concentration higher next to vasculature than in parenchymal regions. Thus, water movement depends on this gradual osmotic gradient along the storage root. Although osmotic pressure rises during development of the root, there is not a concomitant change of turgor, which is maintained relatively constant at about 0.7 MPa (Tomos et al., 1992). This condition would be reached *in vivo* by the accumulation of extracellular solutes, potassium in particular, which osmotically balance the increment of intracellular sucrose; or glycine betaine, which is involved in salt stress responses (for review see Chen and Murata, 2011). Thus, in connected cells water can pass from one cell to the adjacent neighbor through simplastic pathway where the impact of the imposed osmotic gradient is likely to be more attenuated.

### Relevance of PH

Aquaporins from the γ-TIP family are present in the tonoplast (Chrispeels and Maurel, 1994). In the presence of aquaporins, the initial volume changes are attributed to the only movement of water (Pickard, 2008). However, certain TIPs present in the vacuole are inhibited by acidification justifying the consequent reduction of *P*_*f*_ (Sutka et al., 2005; Leitaõ et al., 2012). Here, the nonlinearity observed between the *P*_*f*_/*P*_*s*_ ratio and pH could be attributed to the inhibitory effect of acidification on TIPs. This would explain why vacuoles under different pH conditions do not reach the expected volume as if water were moving freely (Sommer et al., 2007). Moreover, the decrease of *P*_*f*_ alone does not explain the final volume reached by osmosis. Therefore, the relationship with *P*_*s*_ gains importance. As simulated by our model assuming solute and water transport across the tonoplast membrane (*WS* model), the same osmotic gradient generates two simultaneous fluxes, one of water toward inside the vacuole and other of solutes toward outside. Thus, at the same time this solute flux drags water to the outside of the vacuole. This study shows that the *P*_*f*_*/P*_*s*_ ratio determines the volume time course and the final volume reached. Therefore, if *P*_*f*_ is diminished because of aquaporin inhibition, then the dynamics of the volume change is governed by the solute flux.

As defined in the *WS* model, *P*_*f*_ and *P*_*s*_ are two independent parameters. Both *P*_*f*_ and *P*_*s*_ values are obtained for each experiment by a fitting procedure. Therefore, a *P*_*f*_/*P*_*s*_ value is obtained from each experiment, and each *P*_*f*_/*P*_*s*_ ratio is consequence of the vacuole volume time-course in that experiment. The nonlinear pH-dependence of the *P*_*f*_*/P*_*s*_ ratio could be attributed to the regulatory role the vacuole plays in cell physiology. The acidic pH has been reported as physiological. For example, under induced anoxia conditions the cytosolic pH decrease with a half-time of about 30 min, while the vacuolar pH changes with a half-time of about 60–100 min (Kulichikhin et al., 2009). In contrast, pH upper than 8 was not reported *in vivo* (Martinière et al., 2013). Our results show that both *P*_*f*_ and *P*_*s*_ show non-monotonic responses when plotted against pH or proton concentration (Table 1 and Figure S5). However, while *P*_*f*_ shows variations of about one order of magnitude from pH 6.6 to 7.0, *P*_*s*_ shows less variation within the whole pH range (less than one order of magnitude between pH 6.6 and 8.6). The *P*_*f*_ values obtained with the *WS* model in this work and the non-monotonic pH-dependence is in accordance with previously published results obtained with isolated vesicles from beetroot tonoplast (compare Figure S5A with reported results in Figure 5B from Sutka et al., 2005). Other non-monotonic pH dependences have been reported for different transporters present in tonoplast (Davies et al., 1994; Ward and Schroeder, 1994; Hafke et al., 2003), as well as for vacuole stability (Leigh and Branton, 1976). The root beet vacuole can hold its steady state in the pH range between 6.0 and 8.5. The optimal state of the vacuole is maintained at pH 7.5, while a 25% decrease was observed at pH 8.4, and a 30% decrease was observed at pH 6.0 (Leigh and Branton, 1976). Therefore, the response observed at pH 8.6 could combine both the effects on permeability properties as well as on vacuole stability.

In order to address the physiological meaning of *P*_*f*_, *P*_*s*_ and pH, it is important to highlight that since the discovery of aquaporins (Preston et al., 1992) specific pH-modulated AQPs have been reported both in animal and plant membranes (e.g., animal AQP0 in Németh-Cahalan and Hall, 2000; plant PIPs in Tournaire-Roux et al., 2003). These unique associations opened for the first time the question of how water transfers might be affected by pH-dependent permeability changes in a specific membrane. In physiological conditions, the cytosol of a typical plant cell (pH 7.2–7.5; i.e., slightly alkaline) is in between two acidic compartments: the apoplast and the vacuole (pH 4.5–6.0). The cytosol is the only compartment subjected to a tight buffer capacity −20 to 80 mM H^+^ per pH unit- (Felle, 2001, 2005). Highly pH-sensitive reporters are nowadays allowing to quantify pH changes, proving that dynamic changes do occur in many plant cell types and in response to conditions such as salt stress, anoxia, and during growth or developmental stages (Swanson et al., 2011). For instance, acid loading induced in flooded soils has been extensively addressed in the literature, clearly showing that cytosolic acidification is part of a response to anoxia (Felle, 2005). The anoxia and cytosolic acidification has proven to be connected to water fluxes and aquaporins (Tournaire-Roux et al., 2003).

Thus, it is plausible to propose that free protons can be transiently modulated in cell microdomains to act as a signal or a messenger (Felle, 2001) and in consequence modulate membrane permeability. Our work takes into account that *P*_*s*_ should be also included to complete the picture and our simulations are a further step that confirms that ranges are plausible and in agreement with a physiological interpretation.

## Concluding remarks

Nowadays, in plant physiology, integration of ion transport information is at a quantitative stage (Blatt et al., 2014). Here, we tested a number of plausible hypotheses encoded in mathematical models which are constrained by the time course of the external osmolality, making possible to simulate experiments were replacement of the external solution is not instantaneous but gradual, as in ion re-allocation (Mills et al., 1985; Kronzucker and Britto, 2011), or when buffering pH (Kulichikhin et al., 2009). Thus, this approach provides a more realistic situation when simulating experimental conditions. By fitting the models to experimental data, we showed that the vacuole volume changes, under progressively applied osmotic gradients, would not depend on the membrane elastic properties, nor on the non-osmotic volume of the vacuole, but on the water and solute fluxes across the tonoplast. Moreover, we contributed with a phenomenological descriptor (the *P*_*f*_*/P*_*s*_ ratio) which allows linking water and solutes transport with pH. In conclusion, we found that the volume of the vacuole at the steady state is governed by the ratio between the water and solute permeability coefficients (*P*_*f*_/*P*_*s*_), which in turn is determined by pH. Whether this descriptor could be relevant for other scenarios in plant physiology remains to be investigated.

## Author contributions

GA, MS, and VV designed the experiments; VV and MS performed all the experiments; OC developed the models, and VV and MO contributed as well by suggesting and implementing minor modifications; VV, MS, OC, and MO performed the simulations and analysis of results; VV, MS, GA, OC, MO discussed the results and wrote the manuscript.

## Funding

This work was supported by Agencia Nacional de Promoción Científica y Tecnológica [Préstamo BID PICT 2014-3469] to OC, and by the Agencia Nacional para la Promoción Científica y Técnica [Préstamo BID PICT11-2239 and PICT14-0744]; Consejo de Investigaciones Científicas y Técnicas (CONICET) Proyecto de Investigación Plurianual (PIP12-14) and Universidad de Buenos Aires UBACyT14-17 to GA.

### Conflict of interest statement

The authors declare that the research was conducted in the absence of any commercial or financial relationships that could be construed as a potential conflict of interest.
