# Supplementary material for: The Water to Solute Permeability Ratio Governs the Osmotic Volume Dynamics in Beetroot Vacuoles
Source: Front Plant Sci. 2016 Sep 15;7:1388. doi: 10.3389/fpls.2016.01388 (PMC5024706; doi:10.3389/fpls.2016.01388)
Supplement: Supplementary file 1 [file DataSheet1.pdf]

Supplementary Material

The water to solute permeability ratio governs the osmotic volume dynamics in beetroot vacuoles

Victoria Vitali[1†], Moira Sutka[1†], Gabriela Amodeo[1], Osvaldo Chara[2,3], Marcelo Ozu[1,4]*

[1] Departamento de Biodiversidad y Biología Experimental, Facultad de Ciencias Exactas y Naturales, Instituto de Biodiversidad y Biología Experimental y Aplicada (IBBEA), Universidad de Buenos Aires and Consejo Nacional de Investigaciones Científicas y Técnicas (CONICET), Buenos Aires, Argentina.
[2] Instituto de Física de Líquidos y Sistemas Biológicos (IFLYSIB), Consejo Nacional de Investigaciones Científicas y Técnicas(CONICET), Universidad de La Plata, La Plata, Argentina.
[3] Center for Information Services and High Performance Computing (ZIH), TechnischeUniversität Dresden, Dresden, Germany.
[4] Departamento de Fisiología y Biofísica, Facultad de Medicina, Instituto de Fisiología y Biofísica (IFIBIO–Houssay), Universidad de Buenos Aires y Consejo Nacional de Investigaciones Científicas y Técnicas(UBA-CONICET), Buenos Aires, Argentina.

[†] These authors are co-first authors as have equally contributed to this work

* Correspondence: Dr. Marcelo Ozu, Departamento de Biodiversidad y Biología Experimental, Facultad de Ciencias Exactas y Naturales, Instituto de Biodiversidad y Biología Experimental y Aplicada (IBBEA), Universidad de Buenos Aires and Consejo Nacional de Investigaciones Científicas y Técnicas (CONICET).
Int. Güiraldes 2160 (C1428EGA), Buenos Aires, Argentina.
Tel. (54 11) 4576 3300 int. 201.
mozu@bg.fcen.uba.ar

Supplementary Material Index:

Table S1, model variables, parameters and initial conditions — page 2

Figure S1, constant area and variable area simulations — page 2

Figure S2, hypo-osmotic simulations at pH 6.6, 6.8, and 8.6 — page 3

Figure S3, experimental data and the best simulations with the *WS* model — page 3

Figure S4 and **Table A**, the validation of the *WS* model — page 4

Figure S5, P_f and P_s values obtained with the *WS* model under all pH conditions — page 5

Table S2, the comparison of the best fits obtained with each model — page 6

Table S3, the comparison of the best fits obtained with each model — page 7

Table S4, the best parameter values obtained by fitting simulations — page 8

Table S1. Model variables, parameters and initial conditions.

**Model variables**	
V	Vacuole volume (cm^3)
M	Intravacuolar solute content (mol)
**Fixed parameters**	
V_W	Molar volume of water: 18.0 ($cm^3.mol^{-1}$)
A	Vacuole surface area: 5.27 ± 0.98 (10^{-5} cm^2)
C_e	Osmolality of the hypo-osmotic solution: 0.290 ± 0.008 ($Osm.Kg_w^{-1}$) (n = 7)
**Model fitting parameters**	
P_f	Water permeability coefficient ($cm.s^{-1}$)
P_s	Solute permeability coefficient ($cm.s^{-1}$)
ε	Volumetric elastic modulus ($dyn.cm^{-2}$)
V_b	Non-osmotic volume (cm^3)
**Initial conditions**	
V_0	5.39 ± 0.43 (10^{-8} cm^3) (n = 2 to 5 independent experiments involving a total of 4 to14 vacuoles)
M_0	2.64 ± 0.27 (10^{-11}mol) obtained from the initial osmolality of the studied vacuoles $\frac{M}{V}(t = 0) = 0.49 \pm 0.012$ ($Osm.Kg_w^{-1}$) (n = 5)

Figure S1

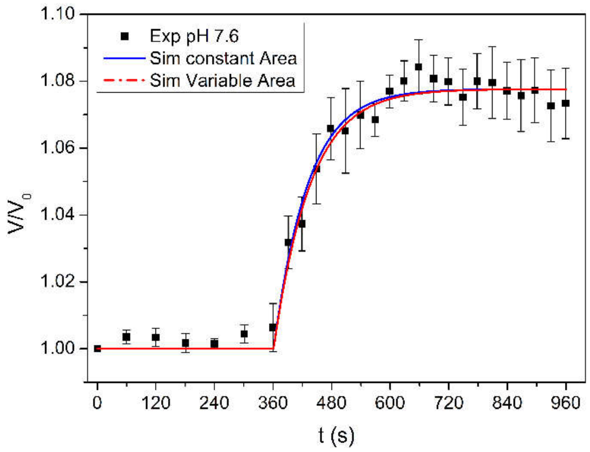

Figure S1. Fitting simulations with the *WS* model considering constant or variable area. The experimental series of pH 7.6 was used to test fitting simulations with constant or variable area. In the first case, the initial area was fixed (5.27 x 10^{-5} cm^2). In the second case the area was modified in each iteration according to its dependence with the volume ($A = 4.836\ V^{2/3}$). Both simulations shown were run with $P_f = 0.017$ $cm.s^{-1}$, and $P_s = 0.00063$ $cm.s^{-1}$ (data of pH 7.6, from Table S4). In average, the best P_f and P_s values (mean ± SEM, n=14) obtained with constant area were (x 10^{-3} $cm.s^{-1}$) 13 ± 3 and 0.47 ± 0.10, respectively. The best P_f and P_s values obtained with variable area were 9 ± 2 and 0.35 ± 0.07, respectively.

Figure S2

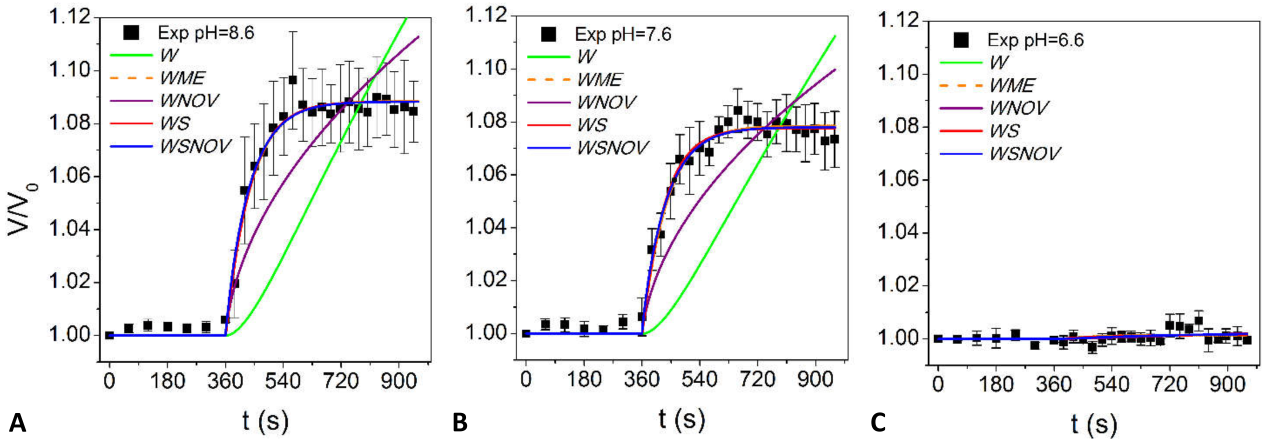

Figure S2. Fitting simulations obtained with the different models tested with published data in different pH conditions. Both *W* and *WNOV* models cannot reproduce the experimental record for pH conditions 7.6 and 8.6. The other three models (*WME*, *WS*, and *WSNOV*) fit close to experimental data and are practically not differentiable by visual inspection. The fitting analysis is shown in Tables S2 and S3, and the parameter values are shown in Table S4.

Figure S3

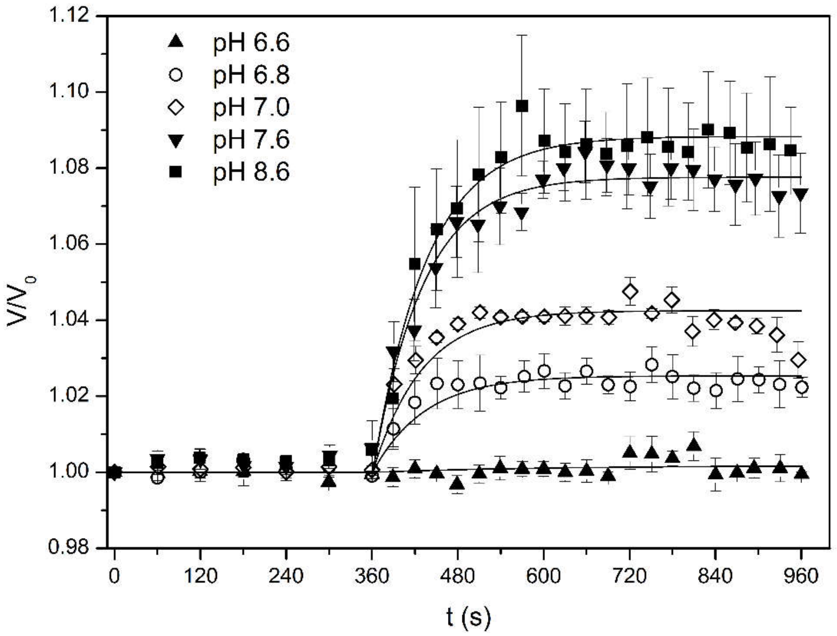

Figure S3. Vacuole osmotic volume changes tested under different pH conditions. Vacuoles were equilibrated with the external solution during 360 s (Iso-osmotic condition: 490 mOsmol.Kg_w^{-1}). Then a hypo-osmotic challenge was imposed by replacing the external solution (Hypo-osmotic condition: 290 mOsmol.Kg_w^{-1}). Full-filled symbols show published results (Amodeo et al., 2002). Open symbols show new experiments of this work. Continuous lines represent the best fit obtained with the *WS* model. Experimental results are shown as mean ± SEM.

Validation of the *WS* model

In order to validate the *WS* model we performed simulations assuming hyper-osmotic conditions. For each model, we adopted P_f and P_s values from the best fitting ranges obtained from the hypo-osmotic conditions at pH 7.6. In the case of the *WS* model, these values are within the ranges (mean ± SEM x 10^{-3}): 9 ± 2 cm.s^{-1} for P_f, and 0.35 ± 0.07 cm.s^{-1} for P_s. Next, we performed experiments exposing vacuoles to external hyper-osmotic conditions and simultaneously determined the time course of external osmolality (Fig. S4A) and vacuole volume (Fig. S4B). The kinetics of the external osmolality was fitted to an exponential function (Fig. S4A) and incorporated into the models. As in hypo-osmotic conditions, both the *WS* and *WSNOV* models reasonably predicts the measured vacuole volume dynamics under hyper-osmotic conditions (Fig. S4B). Therefore, an Akaike's analysis was performed (Table A), which indicate that the *WS* model is the one that better describes the dynamics of vacuoles under hyper-osmotic conditions at pH 7.6 while constrained by the simultaneously measured external osmolarity kinetics.

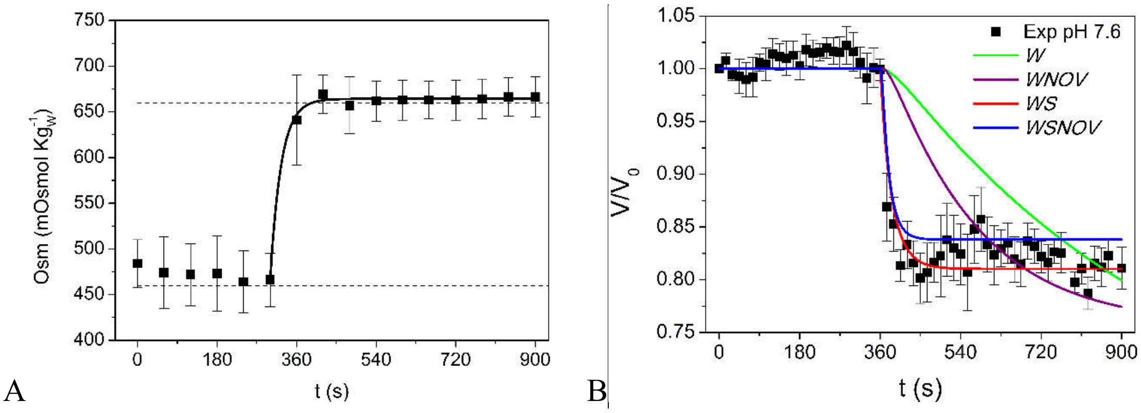

Figure S4. Validation of the *WS* model. A. Experimental determination of the external osmolality kinetics. Dots represent the mean ± SEM of 3 independent experiments. Solid line represents the exponential fit incorporated to the model (R^2>0.99). **B.** Time course of the relative vacuole volume predicted by the four tested mathematical models (*W*, *WNOV*, *WS*, and *WSNOV*, for details on the models, see Section 2 of the main text). Dots and error bars represent the mean ± SEM of 3 independent experiments under the hyper-osmotic conditions whose dynamics is shown in A, at pH 7.6. Solid lines represents the simulations obtained with the models. The exponential fitted in A was incorporated in the model simulations shown in B.

Table A. Comparison of model simulations under hyper-osmotic conditions and pH 7.6. Data presented in the table (P_f, P_s, and $b*V_i=V_b$) were used to simulate the volume time courses of the mean records of experiments at pH 7.6 under hyper-osmotic conditions. R is the result of the fit based on the sum of squares, k is the number of parameters of each model, n is the number of experimental points, AIC is the Akaike's index, and RE is the Evidence Ratio. The ER compares the Akaike's indexes (AIC) of two models and indicates how many times the model with the lowest AIC (the WS model) is preferred over the other. *V_i: initial volume of the vacuole.*

Model	*W*	*WNOV*	*WS*	*WSNOV*
P_f (cm.s^{-1})	2.0 x 10^{-4}	3.0 x 10^{-4}	9.4 x 10^{-3}	1 x 10^{-2}
P_s (cm.s^{-1})	-	-	1.1 x 10^{-4}	1 x 10^{-4}
b	-	0.07	-	0.01
R	0.0037	0,0027	0.00021	0.00039
K	1	2	2	3
N	26	26	26	26
AIC	-141	-147	-213	-194
RE	4.31 x 10^{15}	2.14 x 10^{14}		1.34 x 10^{4}

Figure S5

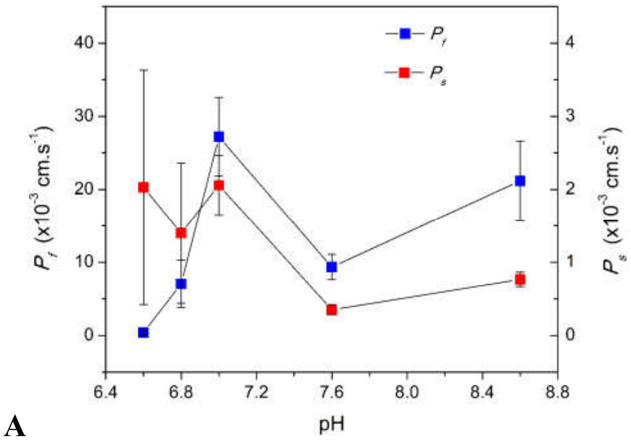

A

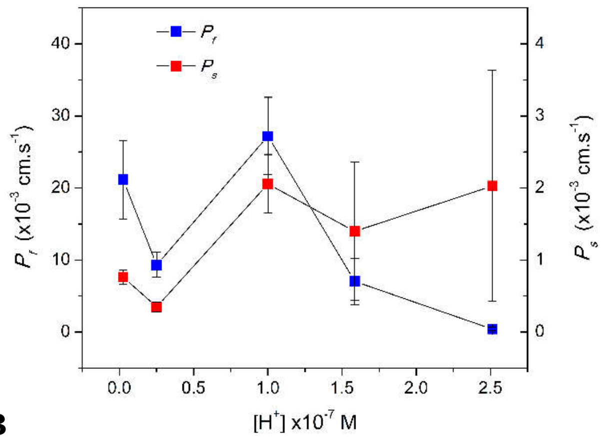

B

Figure S5. Water and solute permeability coefficients obtained with the *WS* model under each pH condition. P_f and P_s values (mean ± SEM) shown in Table 1 are plotted against pH **(A)** or proton concentration **(B)**. While P_f shows variations of about one order of magnitude from pH 6.6 to 7.0, P_s shows less variation within the whole pH range (less than one order of magnitude between pH 6.6 and 8.6). The relationship between P_f and pH is comparable to previously published experimental data (See Fig. 5B in Sutka et al., 2005), reflexing the aquaporins inhibition with acidic pH (at pH 6.6 and 6.8).

Table S2. Comparison of the best fits obtained with the different models tested. R is the result of the fit based on the sum of squares, k is the number of parameters of each model, n is the number of experimental points, and AIC is the Akaike's index. *W*: Water model; *WNOV*: Water and Non-Osmotic Volume model; *WME*: Water and Elasticity Membrane model; *WS*: Water and Solute model; *WSNOV*: Water Solute and Non-Osmotic Volume model.

		**Models**				
**pH**	**Data**	***W***	***WNOV***	***WME***	***WS***	***WSNOV***
**8.6**	**R**	9.57×10^{-4}	2.57×10^{-4}	1.77×10^{-5}	1.69×10^{-5}	1.88×10^{-5}
	**K**	1	2	2	2	3
	**N**	27	27	27	27	27
	**AIC**	-185.54	-216.13	-288.36	-292.18	-284.04
**7.6**	**R**	7.61×10^{-4}	2.02×10^{-4}	1.38×10^{-5}	1.36×10^{-5}	1.38×10^{-5}
	**K**	1	2	2	2	3
	**N**	27	27	27	27	27
	**AIC**	-191.74	-222.66	-295.08	-297.97	-292.32
**7.0**	**R**	2.94×10^{-4}	1.08×10^{-4}	1.35×10^{-5}	8.54×10^{-6}	5.31×10^{-6}
	**K**	1	2	2	2	3
	**N**	26	26	26	26	26
	**AIC**	-209.27	-230.34	-284.41	-296.34	-305.88
**6.8**	**R**	1.07×10^{-4}	4.20×10^{-5}	5.96×10^{-6}	5.43×10^{-6}	3.07×10^{-6}
	**K**	1	2	2	2	3
	**N**	27	27	27	27	27
	**AIC**	-244.80	-265.05	-317.77	-322.86	-332.90
**6.6**	**R**	3.98×10^{-6}	3.98×10^{-6}	4.10×10^{-6}	3.98×10^{-6}	4.04×10^{-6}
	**K**	1	2	2	2	3
	**N**	27	27	27	27	27
	**AIC**	-333.55	-328.66	-327.91	-331.24	-325.49

Table S3. Comparison of the fitting results. The Evidence Ratio (ER) compares the Akaike´s indexes (AIC) of two models and indicates how many times the model with the lowest AIC (Shown in Table S2) is preferred over the other one. Each cell of this double entry Table shows the ER that results from the comparison between two models, which are indicated in the first row and the second column. *W*: Water model; *WNOV*: Water and Non-Osmotic Volume model; *WME*: Water and Elasticity Membrane model; *WS*: Water and Solute model; *WSNOV*: Water Solute and Non-Osmotic Volume model.

	Models	*WME*	*WNOV*	*WS*	*WSNOV*
**pH=8.6**	*W*	2.12 x 10^{22}	4.39 x 10^{6}	1.43 x 10^{23}	2.45 x 10^{21}
	WME		4.83 x 10^{15}	6.74	8.68
	WNOV			4.61 x 10^{23}	5.57 x 10^{14}
	WS				58.53

	Models	*WME*	*WNOV*	*WS*	*WSNOV*
**pH=7.6**	*W*	2.75 x 10^{22}	5.18 x 10^{6}	1.17 x 10^{23}	6.93 x 10^{21}
	WME		5.30 x 10^{15}	4.25	3.96
	WNOV			2.26 x 10^{16}	1.34 x 10^{15}
	WS				16.86

	Models	*WME*	*WNOV*	*WS*	*WSNOV*
**pH=7.0**	*W*	2.07 x 10^{16}	3.76 x 10^{4}	8.07 x 10^{18}	9.52 x 10^{20}
	WME		5.52 x 10^{11}	51.36	4.59 x 10^{4}
	WNOV			2.15 x 10^{14}	2.53 x 10^{16}
	WS				117.92

	Models	*WME*	*WNOV*	*WS*	*WSNOV*
**pH=6.8**	*W*	7.01 x 10^{15}	2.94 x 10^{4}	8.92 x 10^{16}	1.35 x 10^{19}
	WME		2.82 x 10^{11}	12.72	1.92 x 10^{3}
	WNOV			3.59 x 10^{12}	5.43 x 10^{14}
	WS				151.33

	Models	*WME*	*WNOV*	*WS*	*WSNOV*
**pH=6.6**	*W*	16.75	11.49	3.17	56.11
	WME		1.46	5.29	3.35
	WNOV			3.63	4.88
	WS				17.72

Table S4. Parameter values obtained with different models by means of fitting simulations. These results are the best values obtained by fitting simulations performed on the experimental records shown in Figure S2. The non-osmotic volume is expressed as a fraction (b) of the initial volume of the vacuole ($b=V_b/V_i$). For better comparison to reported values, ε is shown in MPa. *W*: Water model; *WNOV*: Water and Non-Osmotic Volume model; *WME*: Water and Elasticity Membrane model; *WS*: Water and Solute model; *WSNOV*: Water Solute and Non-Osmotic Volume model.

		**Models**				
**pH**	**Parameters**	***W***	***WNOV***	***WME***	***WS***	***WSNOV***
**8.6**	P_f **(cm.s^{-1})**	6.60x10^{-5}	1.23 x 10^{-6}	9.90 x 10^{-2}	2.00x10^{-2}	4.30x10^{-4}
	P_s **(cm.s^{-1})**	-	-	-	6.40x10^{-4}	1.90x10^{-5}
	b	-	0.99	-	-	0.23
	ε **(MPa)**	-	-	4.32	-	-
**7.6**	P_f **(cm.s^{-1})**	5.70x10^{-5}	9.80 x 10^{-7}	9.80 x 10^{-2}	1.4 x10^{-2}	1.19 x 10^{-3}
	P_s **(cm.s^{-1})**	-	-	-	5.1 x 10^{-4}	5.10 x 10^{-5}
	b	-	0.99	-	-	0.1
	ε **(MPa)**	-	-	4.95	-	-
**7.0**	P_f **(cm.s^{-1})**	3.00 x 10^{-5}	3.52 x 10^{-7}	9.90 x 10^{-2}	3.3 x 10^{-2}	1.18 x 10^{-5}
	P_s **(cm.s^{-1})**	-	-	-	2.3 x 10^{-3}	2.1 x 10^{-6}
	b	-	0.99	-	-	0.91
	ε **(MPa)**	-	-	9.60	-	-
**6.8**	P_f **(cm.s^{-1})**	1.65 x 10^{-5}	1.50 x 10^{-7}	9.91 x 10^{-2}	7.80 x 10^{-3}	1.20 x 10^{-5}
	P_s **(cm.s^{-1})**	-	-	-	9.60 x 10^{-4}	3.50 x 10^{-6}
	b	-	0.99	-	-	0.84
	ε **(MPa)**	-	-	16.60	-	-
**6.6**	P_f **(cm.s^{-1})**	9.80 x 10^{-7}	9.50 x 10^{-7}	1.00 x 10^{-5}	1.10 x 10^{-3}	1.50 x 10^{-6}
	P_s **(cm.s^{-1})**	-	-	-	2.00 x 10^{-3}	2.70 x 10^{-6}
	b	-	0.01	-	-	0.1
	ε **(Mpa)**	-	-	315	-	-
